# Supplementary material for: Discovery and application of insertion-deletion (INDEL) polymorphisms for QTL mapping of early life-history traits in Atlantic salmon
Source: BMC Genomics. 2010 Mar 8;11:156. doi: 10.1186/1471-2164-11-156 (PMC2838853; doi:10.1186/1471-2164-11-156)
Supplement: Additional file 2 — Information on developed 76 locus single-run INDEL panel in Atlantic salmon. Information on fluorescence labeling, primer concentrations, PCR pooling and links to alignments, INDEL motifs and GENESCAN (Burge and Karlin 1997) predictions of genes/exons are available in html format. [file 1471-2164-11-156-S2.ZIP › Additionalfile2/table13049.html]

Contig Alignment

```
Base	A B C D E F   
1	. . . G G .    
2	. . . C C .    
3	. . . T T .    
4	. . . T T .    
5	G . . G G .    
6	C . . C C .    
7	A . . A A .    
8	A . . A A .    
9	C . . C C .    
10	A . . A A .    
11	G . . G G .    
12	T . . T T .    
13	T . . T T .    
14	G . . G G .    
15	A . . A A .    
16	T . . T T .    
17	T . . T T .    
18	T . . T T .    
19	A . . A A .    
20	T . . T T .    
21	T . . T T .    
22	T . . T T .    
23	T . . T T .    
24	A . . A A .    
25	A . . A A .    
26	C . . C C .    
27	A . . A A .    
28	A . . A A .    
29	T . . T T .    
30	A . . A A .    
31	C . . C C .    
32	C . . C C .    
33	A . . A A .    
34	A . . A A .    
35	A . . A A .    
36	G . . G G .    
37	C . . C C .    
38	A . . A A .    
39	A . . A A .    
40	A . . A A .    
41	A . . A A .    
42	T . . T T .    
43	C . . C C .    
44	T . . T T .    
45	C . . C C .    
46	A . . A A .    
47	C . . C C .    
48	A A A A A .    
49	G G G G G .    
50	A A A A A A    
51	G G G G G G    
52	A A A A A A    
53	C C C C C C    
54	T T T T T T    
55	T T T T T T    
56	T T T T T T    
57	G G G G G G    
58	A A A A A A    
59	T T T T T T    
60	T T T T T T    
61	T T T T T T    
62	A A A A A A    
63	C C C C C C    
64	A A A A A A    
65	T T T T T T    
66	A A A A A A    
67	G G G G G G    
68	A A A A A A    
69	A A A A A A    
70	T T T T T T    
71	G G G G G G    
72	C C C C C C    
73	A A A A A A    
74	A A A A A A    
75	A A A A A A    
76	T T T T T T    
77	A A A A A A    
78	A A A A A A    
79	A A A A A A    
80	T T T T T T    
81	A A A A A A    
82	C C C C C C    
83	A A A A A A    
84	G G G G G G    
85	T T T T T T    
86	A A A A A A    
87	A A A A A A    
88	T T T T T T    
89	C C C C C C    
90	A A A A A A    
91	A A A A A A    
92	A A A A A A    
93	T T T T T T    
94	A A A A A A    
95	G G G G G G    
96	A A A A A A    
97	A A A A A A    
98	T T T T T T    
99	A A A A A A    
100	A A A A A A    
101	A A A A A A    
102	A A A A A A    
103	G G G G G G    
104	G G G G G G    
105	A A A A A A    
106	C C C C C C    
107	A A A A A A    
108	T T T T T T    
109	A A A A A A    
110	A A A A A A    
111	T T T T T T    
112	T T T T T T    
113	A A A A A A    
114	T T T T T T    
115	T T T T T T    
116	T T T T T T    
117	A A A A A A    
118	C C C C C C    
119	A A A A A A    
120	A A A A A A    
121	C C C C C C    
122	A A A A A A    
123	C C C C C C    
124	T T T T T T    
125	T T T T T T    
126	C C C C C C    
127	A A A A A A    
128	A A A A A A    
129	T T T T T T    
130	A A A A A A    
131	A A A A A A    
132	T T T T T T    
133	G G G G G G    
134	C C C C C C    
135	A A A A A A    
136	A A A A A A    
137	A A A A A A    
138	A A A A A A    
139	C C C C C C    
140	C C C C C C    
141	A A A A A A    
142	C C C C C C    
143	A A A A A A    
144	C C C C C C    
145	- A A - - -  2	 indel  
146	- C C - - -  2	 indel  
147	- A A - - -  2	 indel  
148	- C C - - -  2	 indel  
149	A A A A A A    
150	C C C C C C    
151	T T T T T T    
152	G G G G G G    
153	G G G G G G    
154	G G G G G G    
155	A A A A A A    
156	T T T T T T    
157	C C C C C C    
158	T T T T T T    
159	T T T T T T    
160	C C C C C C    
161	A A A A A A    
162	T T T T T T    
163	T T T T T T    
164	G G G G G G    
165	T T T T T T    
166	G G G G G G    
167	G G G G G G    
168	C C C C C C    
169	C C C C C C    
170	A A A A A A    
171	T T T T T T    
172	T T T T T T    
173	T T T T T T    
174	G G G G G G    
175	A A A A A A    
176	A A A A A A    
177	G G G G G G    
178	A A A A A A    
179	C C C C C C    
180	G G G G G G    
181	T T T T T T    
182	A A A A A A    
183	T T T T T T    
184	T T T T T T    
185	A A A A A A    
186	C C C C C C    
187	A A A A A A    
188	T T T T T T    
189	G G G G G G    
190	T T T T T T    
191	G G G G G G    
192	C C C C C C    
193	T T T T T T    
194	G G G G G G    
195	T T T T T T    
196	C C C C C C    
197	C C C C C C    
198	A A A A A A    
199	C C C C C C    
200	T T T T T T    
201	G G G G G G    
202	G G G G G G    
203	T T T T T T    
204	G G G G G G    
205	G G G G G G    
206	G G G G G G    
207	G G G G G G    
208	A A A A A A    
209	A A A A A A    
210	G G G G G G    
211	G G G G G G    
212	A A A A A A    
213	C C C C C C    
214	G G G G G G    
215	G G G G G G    
216	C C C C C C    
217	G G G G G G    
218	A A A A A A    
219	C C C C C C    
220	T T T T T T    
221	G G G G G G    
222	A A A A A A    
223	C C C C C C    
224	A A A A A A    
225	G G G G G G    
226	T T T T T T    
227	A A A A A A    
228	C C C C C C    
229	T T T T T T    
230	G G G G G G    
231	C C C C C C    
232	T T T T T T    
233	T T T T T T    
234	T T T T T T    
235	G G G G G G    
236	T T T T T T    
237	G G G G G G    
238	T T T T T T    
239	C C C C C C    
240	A A A A A A    
241	T T T T T T    
242	G G G G G G    
243	T T T T T T    
244	A A A A A A    
245	G G G G G G    
246	G G G G G G    
247	C C C C C C    
248	A A A A A A    
249	T T T T T T    
250	A A A A A A    
251	C C C C C C    
252	A A A A A A    
253	T T T T T T    
254	C C C C C C    
255	T T T T T T    
256	T T T T T T    
257	T T T T T T    
258	T T T T T T    
259	A A A A A A    
260	G G G G G G    
261	C C C C C C    
262	C C C C C C    
263	C C C C C C    
264	A A A A A A    
265	A A A A A A    
266	C C C C C C    
267	A A A A A A    
268	T T T T T T    
269	C C C C C C    
270	T T T T T T    
271	G G G G G G    
272	A A A A A A    
273	C C C C C C    
274	T T T T T T    
275	A A A A A A    
276	C C C C C C    
277	A A A A A A    
278	A A A A A A    
279	C C C C C C    
280	T T T T T T    
281	A A A A A A    
282	C C C C C C    
283	C C C C C C    
284	C C C C C C    
285	A A A A A A    
286	A A A A A A    
287	C C C C C C    
288	C C C C C C    
289	G G G G G G    
290	T T T T T T    
291	A A A A A A    
292	A A A A A A    
293	C C C C C C    
294	A A A A A A    
295	C C C C C C    
296	G G G G G G    
297	A A A A A A    
298	T T T T T T    
299	A A A A A A    
300	T T T T T T    
301	A A A A A A    
302	A A A A A A    
303	C C C C C C    
304	A A A A A A    
305	G G G G G G    
306	A A A A A A    
307	C C C C C C    
308	G G G G G G    
309	T T T T T T    
310	C C C C C C    
311	A A A A A A    
312	C C C C C C    
313	T T T T T T    
314	T T T T T T    
315	C C C C C C    
316	A A A A A A    
317	A A A A A A    
318	C C C C C C    
319	A A A A A A    
320	C C C C C C    
321	T T T T T T    
322	C C C C C C    
323	T T T T T T    
324	T T T T T T    
325	C C C C C C    
326	A A A A A A    
327	T T T T T T    
328	G G G G G G    
329	T T T T T T    
330	C C C C C C    
331	C C C C C C    
332	C C C C C C    
333	G G G G G G    
334	T T T T T T    
335	C C C C C C    
336	A A A A A A    
337	A A A A A A    
338	C C C C C C    
339	A A A A A A    
340	C C C C C C    
341	A A A A A A    
342	G G G G G G    
343	A A A A A A    
344	G G G G G G    
345	T T T T T T    
346	A A A A A A    
347	C C C C C C    
348	C C C C C C    
349	C C C C C C    
350	A A A A A A    
351	G G G G G G    
352	T T T T T T    
353	A A A A A A    
354	T T T T T T    
355	C C C C C C    
356	G G G G G G    
357	A A A A A A    
358	G G G G G G    
359	C C C C C C    
360	T T T T T T    
361	A A A A A A    
362	G G G G G G    
363	A A A A A A    
364	T T T T T T    
365	G G G G G G    
366	T T T T T T    
367	T T T T T T    
368	A A A A A A    
369	G G G G G G    
370	A A A A A A    
371	T T T T T T    
372	T T T T T T    
373	G G G G G G    
374	A A A A A A    
375	A A A A A A    
376	A A A A A A    
377	C C C C C C    
378	A A A A A A    
379	G G G G G G    
380	A A A A A A    
381	C C C C C C    
382	A A A A A A    
383	A A A A A A    
384	T T T T T T    
385	T T T T T T    
386	T T T T T T    
387	G G G G G G    
388	T T T T T T    
389	C C C C C C    
390	G G G G G G    
391	G G G G G G    
392	T T T T T T    
393	C C C C C C    
394	G G G G G G    
395	A A A A A A    
396	A A A A A A    
397	A A A A A A    
398	A A A A A A    
399	A A A A A A    
400	C C C C C C    
401	A A A A A A    
402	A A A A A A    
403	G G G G G G    
404	A A A A A A    
405	G G G G G G    
406	A A A A A A    
407	A A A A A A    
408	A A A A A A    
409	A A A A A A    
410	A A A A A A    
411	A A A A A A    
412	A A A A A A    
413	C A A C C C  2	 transversion  
414	C C C C C C    
415	C C C C C C    
416	C C C C C C    
417	C C C C C C    
418	A A A A A A    
419	A A A A A A    
420	G G G G G G    
421	T T T T T T    
422	T T T T T T    
423	A A A A A A    
424	A A A A A A    
425	A A A A A A    
426	A A A A A A    
427	C C C C C C    
428	A A A A A A    
429	A A A A A A    
430	A A A A A A    
431	A A A A A A    
432	A A A A A A    
433	T T T T T T    
434	C C C C C C    
435	A A A A A A    
436	C C C C C C    
437	A A A A A A    
438	G G G G G G    
439	A A A A A A    
440	C C C C C C    
441	T T T T T T    
442	G G G G G G    
443	A A A A A A    
444	T T T T T T    
445	A A A A A A    
446	T T T T T T    
447	A A A A A A    
448	T T T T T T    
449	T T T T T T    
450	G G G G G G    
451	C C C C C C    
452	A A A A A A    
453	A A A A A A    
454	C C C C C C    
455	A A A A A A    
456	G G G G G G    
457	T T T T T T    
458	A A A A A A    
459	C C C C C C    
460	A A A A A A    
461	A A A A A A    
462	T T T T T T    
463	G G G G G G    
464	T T T T T T    
465	G G G G G G    
466	A A A A A A    
467	T T T T T T    
468	T T T T T T    
469	C C C C C C    
470	T T T T T T    
471	C C C C C C    
472	A A A A A A    
473	A A A A A A    
474	T T T T T T    
475	G G G G G G    
476	A A A A A A    
477	A A A A A A    
478	G G G G G G    
479	A A A A A A    
480	G G G G G G    
481	T T T T T T    
482	G G G G G G    
483	C C C C C C    
484	A A A A A A    
485	T T T T T T    
486	T T T T T T    
487	A A A A A A    
488	T T T T T T    
489	C C C C C C    
490	T T T T T T    
491	C C C C C C    
492	T T T T T T    
493	G G G G G G    
494	T T T T T T    
495	G G G G G G    
496	G G G G G G    
497	T T T T T T    
498	G G G G G G    
499	G G G G G G    
500	A A A A A A    
501	C C C C C C    
502	A A A A A A    
503	A A A A A A    
504	G G G G G G    
505	G G G G G G    
506	A A A A A A    
507	C C C C C C    
508	A A A A A A    
509	A A A A A A    
510	A A A A A A    
511	C C C C C C    
512	T T T T T T    
513	C C C C C C    
514	A A A A A A    
515	A A A A A A    
516	T T T T T T    
517	G G G G G G    
518	A A A A A A    
519	G G G G G G    
520	T T T T T T    
521	G G G G G G    
522	T T T T T T    
523	G G G G G G    
524	A A A A A A    
525	. C C C C C    
526	. A A A A A    
527	. A A A A A    
528	. C C C C C    
529	. C C C C C    
530	. A A A A A    
531	. A A A A A    
532	. C C C C C    
533	. C C C C C    
534	. C C C C C    
535	. C T T T T    
536	. . G G G G    
537	. . A A A A    
538	. . G G G G    
539	. . T T T T    
540	. . G G G G    
541	. . G G G G    
542	. . T T T T    
543	. . G G G G    
544	. . C C C C    
545	. . T T T T    
546	. . G G G G    
547	. . T T T T    
548	. . G G G G    
549	. . A A A A    
550	. . C C C C    
551	. . C C C C    
552	. . C C C C    
553	. . T T T T    
554	. . G G G G    
555	. . A A A A    
556	. . A A A A    
557	. . A A A A    
558	. . C C C C    
559	. . T T T T    
560	. . G G G G    
561	. . G G G G    
562	. . C C C C    
563	. . A A A A    
564	. . G G G G    
565	. . G G G G    
566	. . T T T T    
567	. . C C C C    
568	. . C G G G    
569	. . . C C C    
570	. . . A A A    
571	. . . C C C    
572	. . . C C C    
573	. . . C C C    
574	. . . A A A    
575	. . . C C C    
576	. . . C C C    
577	. . . C C C    
578	. . . C C C    
579	. . . T T T    
580	. . . G G G    
581	. . . A A A    
582	. . . G G G    
583	. . . A A A    
584	. . . A A A    
585	. . . G G G    
586	. . . A A A    
587	. . . A A A    
588	. . . C C C    
589	. . . A A A    
590	. . . T T T    
591	. . . A A A    
592	. . . A A A    
593	. . . A A A    
594	. . . G G G    
595	. . . G G G    
596	. . . T T T    
597	. . . C C C    
598	. . . T T T    
599	. . . T T T    
600	. . . T T T    
601	. . . G G G    
602	. . . C C C    
603	. . . A A A    
604	. . . C C C    
605	. . . C C C    
606	. . . A A A    
607	. . . T T T    
608	. . . C C C    
609	. . . A A A    
610	. . . A A A    
611	. . . A A A    
612	. . . T T T    
613	. . . T T T    
614	. . . A A A    
615	. . . C C C    
616	. . . T T T    
617	. . . A A A    
618	. . . A A A    
619	. . . A A A    
620	. . . C C C    
621	. . . T T T    
622	. . . A A A    
623	. . . A A A    
624	. . . A A A    
625	. . . G G G    
626	. . . A A A    
627	. . . A A A    
628	. . . T T T    
629	. . . G G G    
630	. . . A A A    
631	. . . A A A    
632	. . . A A A    
633	. . . A A A    
634	. . . T T T    
635	. . . G G G    
636	. . . C C C    
637	. . . C C C    
638	. . . T T T    
639	. . . G G G    
640	. . . C C C    
641	. . . T T T    
642	. . . T T T    
643	. . . C C C    
644	. . . C C C    
645	. . . C C C    
646	. . . . C C    
647	. . . . A A    
648	. . . . C C    
649	. . . . C C    
650	. . . . A A    
651	. . . . C C    
652	. . . . C C    
653	. . . . T T    
654	. . . . T T    
655	. . . . A A    
656	. . . . G G    
657	. . . . T T    
658	. . . . C C    
659	. . . . A A    
660	. . . . T T    
661	. . . . A A    
662	. . . . T T    
663	. . . . T T    
664	. . . . T T    
665	. . . . G G    
666	. . . . A A    
667	. . . . T T    
668	. . . . G G    
669	. . . . T T    
670	. . . . C C    
671	. . . . T T    
672	. . . . T T    
673	. . . . T T    
674	. . . . G G    
675	. . . . G G    
676	. . . . A A    
677	. . . . T T    
678	. . . . T T    
679	. . . . T T    
680	. . . . G G    
681	. . . . G G    
682	. . . . G G    
683	. . . . C C    
684	. . . . A A    
685	. . . . G G    
686	. . . . G G    
687	. . . . T T    
688	. . . . T T    
689	. . . . C C    
690	. . . . A A    
691	. . . . G G    
692	. . . . C C    
693	. . . . A A    
694	. . . . A A    
695	. . . . C C    
696	. . . . T T    
697	. . . . T T    
698	. . . . A A    
699	. . . . A A    
700	. . . . A A    
701	. . . . G G    
702	. . . . T T    
703	. . . . G G    
704	. . . . C C    
705	. . . . C C    
706	. . . . T T    
707	. . . . T T    
708	. . . . C C    
709	. . . . G G    
710	. . . . C C    
711	. . . . T T    
712	. . . . T T    
713	. . . . G G    
714	. . . . C C    
715	. . . . T T    
716	. . . . C C    
717	. . . . A A    
718	. . . . C C    
719	. . . . C C    
720	. . . . A A    
721	. . . . G G    
722	. . . . A A    
723	. . . . G G    
724	. . . . C C    
725	. . . . C C    
726	. . . . A A    
727	. . . . T T    
728	. . . . T T    
729	. . . . A A    
730	. . . . T T    
731	. . . . T T    
732	. . . . T T    
733	. . . . T T    
734	. . . . G G    
735	. . . . G G    
736	. . . . C C    
737	. . . . C C    
738	. . . . T T    
739	. . . . C C    
740	. . . . T T    
741	. . . . T T    
742	. . . . T T    
743	. . . . T T    
744	. . . . T T    
745	. . . . G G    
746	. . . . G G    
747	. . . . T T    
748	. . . . T T    
749	. . . . T T    
750	. . . . T T    
751	. . . . T T    
752	. . . . T T    
753	. . . . T T    
754	. . . . T T    
755	. . . . A A    
756	. . . . A A    
757	. . . . C C    
758	. . . . A A    
759	. . . . A A    
760	. . . . A A    
761	. . . . C C    
762	. . . . A A    
763	. . . . A A    
764	. . . . T T    
765	. . . . T T    
766	. . . . C C    
767	. . . . T T    
768	. . . . C C    
769	. . . . A A    
770	. . . . C C    
771	. . . . A A    
772	. . . . A A    
773	. . . . C C    
774	. . . . A A    
775	. . . . G G    
776	. . . . A A    
777	. . . . C C    
778	. . . . T T    
779	. . . . C C    
780	. . . . C C    
781	. . . . A A    
782	. . . . T T    
783	. . . . T T    
784	. . . . T T    
785	. . . . A A    
786	. . . . A A    
787	. . . . A A    
788	. . . . A A    
789	. . . . A A    
790	. . . . A A    
791	. . . . A A    
792	. . . . A A    
793	. . . . C C    
794	. . . . C C    
795	. . . . T T    
796	. . . . C C    
797	. . . . G G    
798	. . . . T T    
799	. . . . G G    
800	. . . . C C
```
